# Mutational spectrum of Barrett's stem cells suggests paths to initiation of a precancerous lesion

**DOI:** 10.1038/ncomms10380

**Published:** 2016-01-19

**Authors:** Yusuke Yamamoto, Xia Wang, Denis Bertrand, Florian Kern, Ting Zhang, Marcin Duleba, Supriya Srivastava, Chiea Chuen Khor, Yuanyu Hu, Lane H. Wilson, Hagen Blaszyk, Daniil Rolshud, Ming Teh, Jianjun Liu, Brooke E. Howitt, Matthew Vincent, Christopher P. Crum, Niranjan Nagarajan, Khek Yu Ho, Frank McKeon, Wa Xian

**Affiliations:** 1 Division of Molecular and Cellular Medicine, National Cancer Center Research Institute, Tokyo 104-0045, Japan; 2 Department of Biology and Biochemistry, University of Houston, Houston, Texas 77204, USA; 3 Department of Computational & Systems Biology, Genome Institute of Singapore, A-STAR, Singapore 138672, Singapore; 4 Department of Pathology, National University Heath System, Singapore 119228, Singapore; 5 Department of Genetics and Developmental Biology, University of Connecticut Health Center, Farmington, Connecticut 06030, USA; 6 Digestive Health Center, Maine Medical Center, South Portland, Maine 04102, USA; 7 Department of Pathology, Brigham and Women's Hospital, Boston, Massachusetts 02115, USA; 8 Ocata Therapeutics, Inc., Marlborough, Massachusetts 01752, USA; 9 Department of Medicine, National University of Singapore, Singapore 119228, Singapore; 10 Department of Microbiology, National University of Singapore, Singapore 119228, Singapore; 11 MultiClonal Therapeutics, Inc., Farmington, Connecticut 06030, USA; 12 Center for Stem Cell & Regenerative Medicine, The University of Texas Health Science Center at Houston, Houston, Texas 77030, USA; 13 Department of Biochemistry and Molecular Biology, The University of Texas McGovern Medical School, Houston, Texas 77030, USA

## Abstract

The precancerous lesion known as Barrett's oesophagus can evolve to oesophageal adenocarcinoma in decades-long processes of regenerative growth. Here we report the isolation and propagation of distinct, patient-matched stem cells of Barrett's, gastric and oesophageal epithelia that yield divergent tumour types following *in vitro* transformation and xenografting. Genomic analyses reveal a broad mutational spectrum unique to Barrett's stem cells that likely reflects their risk for oncogenesis. Remarkably, 25% of cases show no cancer-related genomic changes, suggesting that Barrett's initiates without driver mutations. Most cases, however, sustain patterns of deletions almost identical to adenocarcinoma though tumour-associated gene amplifications were absent. Notably, those suspected of low-grade dysplasia have p53 mutations or undergo amplifications of proto-oncogenes and receptor tyrosine kinases, implicating these events in lethal transitions. Our findings suggest paths for the initiation and progression of Barrett's and define a discrete stem cell underlying its regenerative growth whose eradication could prevent oesophageal adenocarcinoma.

## Abstract (web summary)

Barrett's oesophagus is a precancerous intestinal metaplasia that can progress to oesophageal adenocarcinoma. In this study, the authors isolate and characterize human Barrett's stem cells and identify a specific genomic pedigree that supports the potential role of these cells as precursors of oesophageal adenocarcinoma.

## Graphical abstract

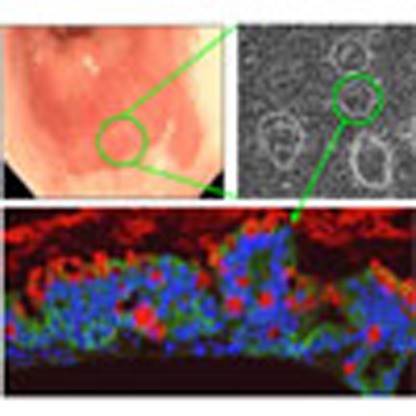

Oesophageal adenocarcinoma (EAC) is a highly lethal cancer whose incidence has quadrupled in the past four decades^1^,^2^,^3^. Efforts at chemotherapy and surgical resection have not appreciably altered survival rates for this cancer, and therefore much hope is placed on early detection and therapeutic eradication of advanced stages of Barrett's oesophagus, a precancerous intestinal metaplasia in the distal oesophagus, before it can progress to EAC^1^,^2^,^4^,^5^,^6^,^7^. As with precursor lesions in other epithelial cancer precursors^8^,^9^, Barrett's is thought to predate the appearance of adenocarcinoma by one or more decades and overall progresses to cancer at a rate of 0.2–1% per year^10^. Efforts to preempt the progression of dysplastic Barrett's to adenocarcinoma employ non-specific technologies such as radiofrequency ablation to remove surface epithelia harbouring this intestinal metaplasia^11^. While remarkably effective especially in focused centres, recurrences of Barrett's and dysplasia, as well as the emergence of EAC remain problematic^12^,^13^,^14^. These recurrences may be due to the survival of hypothetical Barrett's stem cells in post-ablation mucosa, suggesting potential advantages of specifically targeting this stem cell population as part of a broader therapeutic approach to reducing rates of EAC. The existence of stem cells underlying the regenerative growth of Barrett's oesophagus, or indeed any other precursor lesion of an epithelial cancer, has not been established. Though the existence of stem cells from normal columnar epithelia such as intestine have been firmly demonstrated by multiple albeit indirect criteria *in vivo* and *in vitro* especially organoids^15^, until recently there has been no technology that captures and maintains these stem cells in their most immature form.

The present study exploits technology^16^ we originally developed to enable the capture of undifferentiated or ‘ground state' intestinal stem cells to the problem of Barrett's oesophagus. In particular, we used this technology to isolate ground state stem cells from patient-matched, endoscopic biopsies of oesophageal, Barrett's, and stomach and to establish representative, single-cell-derived clonal lines or ‘pedigrees' from each. We show that these pedigrees from the oesophagus, stomach and Barrett's possess all of the canonical features of stem cells including (1) long-term self-renewal, (2) multipotent differentiation and (3) absolute commitment to the respective lineages from which they were derived. Extensive analyses of the oesophageal, stomach and Barrett's stem cells from all 12 Barrett's cases, as well as the cognate epithelia derived from them, demonstrate that Barrett's stem cells are distinct from those of the oesophagus or the stomach. Moreover, mutational and transformation analyses of these distinct stem cell types provide insights to the origin, progression and possible therapeutic strategies for elimination of the Barrett's lesion.

## Results

### Clonogenic cells from Barrett's patients

**Figure 1:**
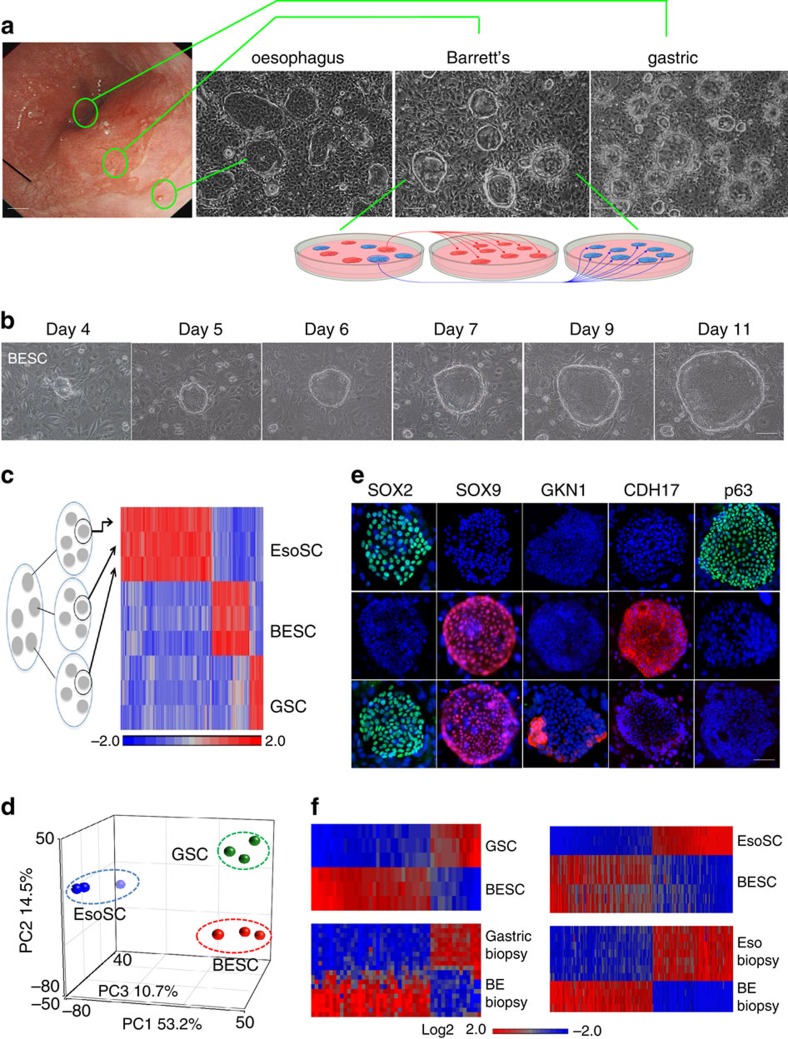
Stem cells from the distal oesophagus of Barrett's patient. (**a**) Left, Endoscopic image of distal oesophagus of a Barrett's patient and approximate locations of biopsies. Right, phase contrast images of typical colonies derived from biopsies. Inset diagram, schematic of cloning process to yield Barrett's or oesophageal stem cell pedigrees from mixed population from Barrett's biopsies. Scale bar, 150 μm. *n*=12 biological replicates. (**b**) Colony formation from a single stem cell over indicated days in culture. Scale bar, 50 μm. (**c**) Heatmap of differential gene expression of three independent pedigrees from each of oesophageal (EsoSC), Barrett's (BESC) and stomach (GSC) stem cells. (**d**) PCA of expression data of three pedigrees each of oesophageal, Barrett's and stomach stem cells. (**e**) Representative immunofluorescence labelling of colonies of EsoSC, BESC and GSC with antibodies to the indicated marker proteins (green or red) with nuclei counterstained with DAPI (blue). Scale bar, 50 μm. *n*=12 biological replicates. (**f**) Gene expression heatmap comparing those differentially expressed between immature GSC (*n*=3) and Barrett's stem cells (BESC; *n*=3) with those from endoscopic gastric biopsy and Barrett's biopsies (BE-biopsy) (GSE34619) (ref. 20).

Endoscopic mucosal biopsies were obtained from 12 Barrett's patients at sites identified as oesophagus, Barrett's and anterior stomach (Fig. 1a). Colonies arose 1 week after plating single cell suspensions of these 1 mm biopsies onto lawns of irradiated 3T3 cells in SCM-68 media known to support immature, epithelial stem cells^16^,^17^ (Fig. 1a and Supplementary Fig. 1a). While colonies from the oesophageal and stomach biopsies were positive for antibodies to keratin 5 (Krt5) or gastrokine 1 (Gkn1), respectively, those from Barrett's yielded mixtures of Krt5-positive clones typical of the oesophagus and ones that expressed the intestinal marker cadherin 17 (Cdh17) (Supplementary Fig. 1b). To separate these two populations of colonies derived from the Barrett's biopsies, we sampled and expanded multiple single colonies as independent pedigrees^18^ (Fig. 1a (schematic) and Supplementary Fig. 1b). Reprobing these independent pedigree lines with the same antibodies showed that the original Barrett's biopsies harboured two distinct types of clonogenic (that is, the ability in an iterative fashion to form colonies of immature cells from a single cell) cells marked by committed expression of either Krt5 or Cdh17 (Supplementary Fig. 1b). Whole-genome expression analysis of three independent, single-cell-derived pedigrees of the Krt5+, Cdh17+ and Gkn1+ immunophenotypes revealed distinct expression profiles consisting of 100–200 genes including those reported in the respective epithelia (Fig. 1c and Supplementary Fig. 1c). Principle component analysis (PCA) of expression data sets showed that the Krt5+, Cdh17+ and Gkn1+ pedigrees occupy unique expression spaces, further supporting the notion that oesophageal, gastric and Barrett's stem cells had stable and distinguishable properties (Fig. 1d). This PCA map also indicated that the stomach and Barrett's stem cells are considerably ‘closer' than each is to the oesophageal stem cells, a result consistent with the fact that both the stomach and Barrett's are columnar epithelia, whereas the oesophageal stem cells are of the general class of stratified epithelia. Consistent with these distinct gene expression profiles, antibodies to Sox2, Sox9, Cdh17, Gkn1 also revealed differential staining patterns (Fig. 1e and Supplementary Fig. 1d). Significantly, the expression profiles of these distinct classes of stem cells from oesophageal, Barrett's and stomach epithelia were mirrored by both marker staining in biopsies (Supplementary Fig. 1e,f) as well as gene expression differences between whole-endoscopic biopsies of oesophagus, Barrett's and stomach epithelia generated by other investigators^19^,^20^ (Fig. 1f). Lastly, we estimate that the clonogenic cells represent ∼1:1,000–1:2,000 of the total epithelial cells recovered from these endoscopic biopsies, ratios similar to those we recently reported for the human intestine and colon^16^.

### Clonogenic cells of distal oesophagus are true stem cells

**Figure 2:**
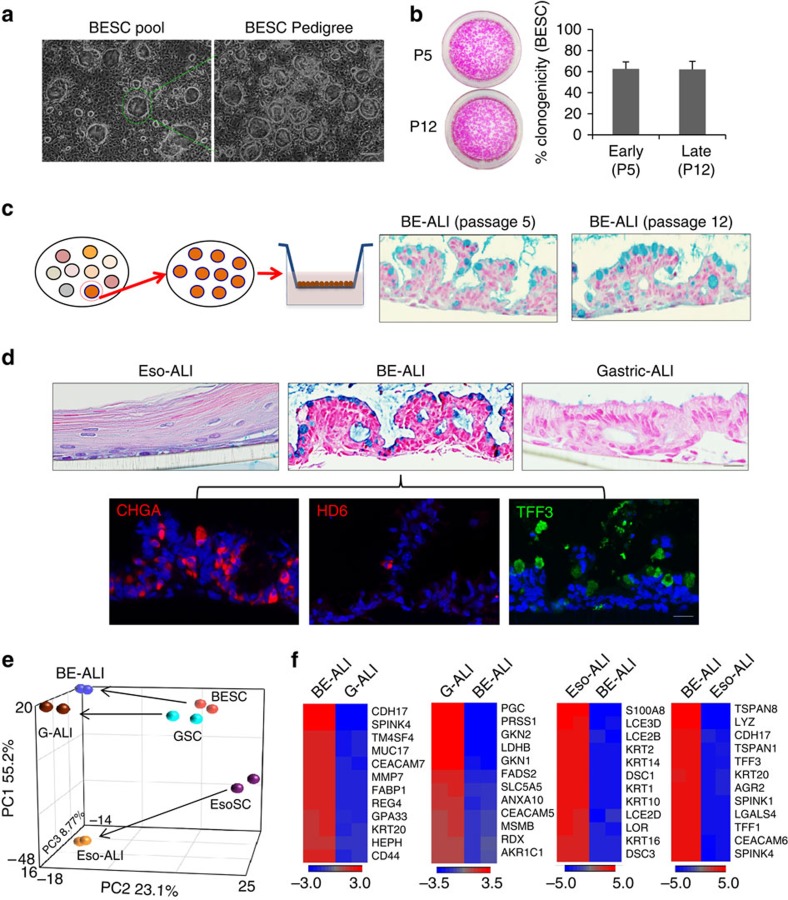
Barrett's stem cell pedigree recapitulates intestinal metaplasia. (**a**) Derivation of stem cell pedigree from pool of colonies (left) by selecting individual colony for replating and expansion (right). Scale bar, 200 μm. *n*=12 biological replicates. (**b**) Clonogenicity assay for BESC at passage 5 and passage 12 determined by plating 2,000 cells and counting colony formation following Rhodamine red staining. Histogram depicting colony counts. Error bars, s.d. *n*=3 biological replicates. (**c**) Left, schematic diagram of induced differentiation of stem cell pedigree in ALI culture. Right, histological section of differentiated p5 and p12 Barrett's stem cells with Alcian blue-positive goblet cells. Scale bar, 100 um. *n*=12 biological replicates. (**d**) Upper, Alcian blue staining of ALI culture differentiation of EsoSC, BESC and GSC, bottom, immunostainings of section of BESC-derived epithelium following ALI differentiation showed distribution of multiple types of cells positive for CHGA (endocrine cells), HD6 (Paneth cells) and TFF3 (goblet cells). Scale bar, 50 μm. *n*=12 biological replicates. (**e**) PCA of whole-genome expression data sets from EsoSC, BESC and GSC before and after ALI differentiation as indicated. (**f**) Expression heatmap of differentially expressed genes in ALI cultures as indicated.

Adult stem cells are typically defined as having the capacity for long-term self-renewal and multipotent differentiation. These properties can be readily tested in single-cell-derived stem cell pedigrees (Fig. 2a) of oesophagus, Barrett's or stomach; all could be grown continuously for months via serial passaging while maintaining their immature, undifferentiated appearances, as well as high rates of clonogenicity (Fig. 2b). As such, the cells in these pedigrees display long-term self-renewal capacity of at least 60 divisions typical of true stem cells in contrast to short-lived progenitor cells^21^. Despite these prolonged periods of continuous cell division, these stem cells maintained their commitment to their respective tissues on differentiation in air–liquid interface (ALI) cultures^16^,^18^ (Fig. 2c and Supplementary Fig. 2a). Consistent with the squamous properties of the oesophageal epithelium, the oesophageal stem cell pedigrees differentiated in ALI cultures to mature squamous cells like those of the epidermis (Fig. 2d). In contrast, Barrett's pedigrees differentiated to columnar epithelia with pathognomonic, Alcian blue-staining goblet cells typical of Barrett's biopsies (Fig. 2c,d and Supplementary Fig. 2b). In addition, abundant chromogranin A (ChgA)-positive endocrine cells were detected in both biopsies of Barrett's and in the ALI-generated epithelium (Fig. 2d and Supplementary Fig. 2b), as were TFF3+ epithelial cells, and rare, α-defensin 6-positive (HD6+) Paneth cells (Fig. 2d and Supplementary Fig. 2b). Anterior stomach stem cells, as expected, differentiated to an epithelium without Alcian blue-staining goblet cells (Fig. 2d). We next analysed the gene expression in the *in vitro*-differentiated epithelia from these stem cells. From a PCA of 1,031 genes differentially expressed in these ALI cultures (Fig. 2e), the Barrett's and stomach stem cells and their corresponding ALI-differentiated epithelia show similar patterns as expected for columnar epithelia, while the oesophageal stem cells and their differentiated epithelia occupy different expression spaces consistent with their squamous properties. However, *in vitro*-differentiated epithelia from these stem cells expressed distinct gene expression signature that is consistent with their tissue origin (Fig. 2f). Together, these data support the conclusion that we have identified and cloned distinct stem cells of the oesophagus, stomach and Barrett's epithelia.

### Barrett's stem cells are distinct from stomach and intestine

**Figure 3:**
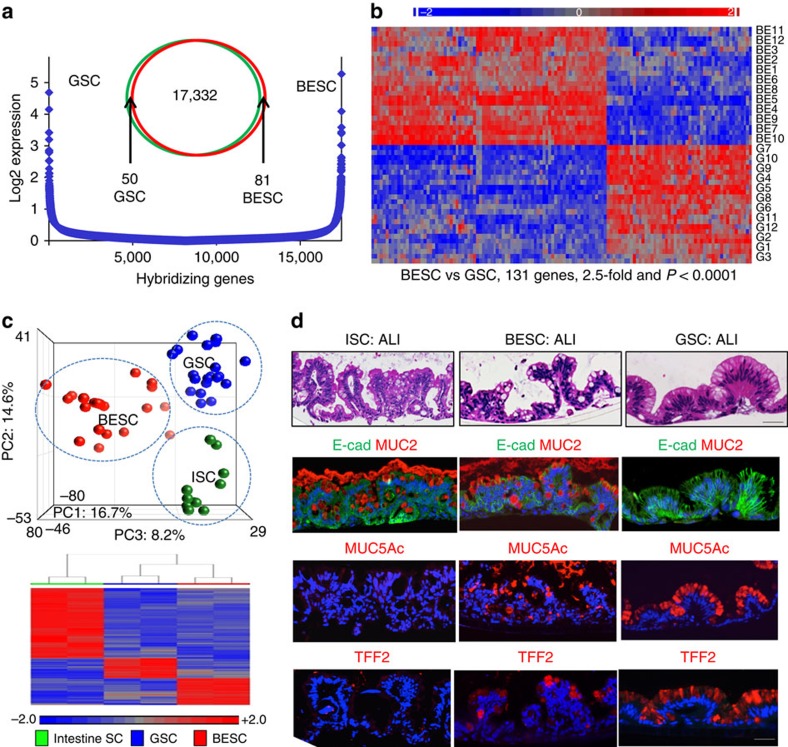
Stem cells of the gastroesophageal junction. (**a**) Comparison of gene expression microarray data from Barrett's and gastric stem cells by Log2 expression distribution and Venn diagrams. (**b**) Gene expression heatmap of patient-matched BESC and GSC pedigrees showing relative expression of the 131 most differentially expressed genes between BESC and GSC (>2.5-fold, *P*<0.0001). (**c**) Top, PCA of whole-genome expression microarray data of BESC, GSC and stem cells of human fetal intestine (ISC)^16^. Bottom, heatmap of differentially expressed genes between BESC, GSC and ISC. (**d**) Histological sections of ALI-differentiated ISC, BESC and GSC stained with (from top) H&E, E-cadherin (E-cad) and mucin 2 (Muc2) antibodies, mucin 5AC (Muc5AC) antibodies and Trefoil factor 2 (TFF2) antibodies. Scale bar, 50 μm. *n*=12 biological replicates.

The relationship between Barrett's intestinal metaplasia, intestine and stomach epithelia has been the subject of much discussion^22^,^23^,^24^,^25^. To specifically address this question, we compared patient-matched stem cells from Barrett's and stomach epithelia from 12 patients with clinical Barrett's without evidence of high-grade dysplasia, which revealed distinguishing expression signatures consisting of ∼131 genes (2.5-fold, *P*<0.0001, Student's *t*-test; Fig. 3a,b, Supplementary Fig. 3, Supplementary Table 1 and Supplementary Data 1). This degree of differential gene expression is similar to that found between stem cells from the upper and lower airways^18^ or between stem cells of the duodenum and jejunum^16^. Extending this analysis, we also found that Barrett's stem cells had whole-genome expression profiles that could be readily distinguished from those of both stomach and intestine by PCA and by gene expression heatmaps (Fig. 3c and Supplementary Figs 4 and 5). These distinctions at the level of stem cell gene expression were also reflected in the epithelia produced by each of these stem cells in ALI cultures (Fig. 3d). For instance, both the intestinal and Barrett's stem cells formed a columnar epithelium complete with goblet cells, whereas the stomach stem cells, as expected, lacked goblet cells. Consistently, both the intestinal and Barrett's stem cells expressed Muc2 typical for goblet cells, while the stomach epithelium did not. However, Barrett's expresses mucin 5AC (Muc5AC), a mucopolysaccharide usually associated with gastric and even airway epithelia, whereas the epithelium derived from the intestinal ileum stem cells does not (Fig. 3d). Similar results were obtained with TFF2, a marker typically associated with gastric epithelium, which was expressed in Barrett's but not the intestinal epithelium (Fig. 3d). Together these data mark Barrett's oesophagus as an unusual columnar epithelium that is clearly distinct from both the stomach as well as intestine despite its characterization as ‘intestinal metaplasia'.

### Tumours from transformed Barrett's and oesophageal stem cells

**Figure 4:**
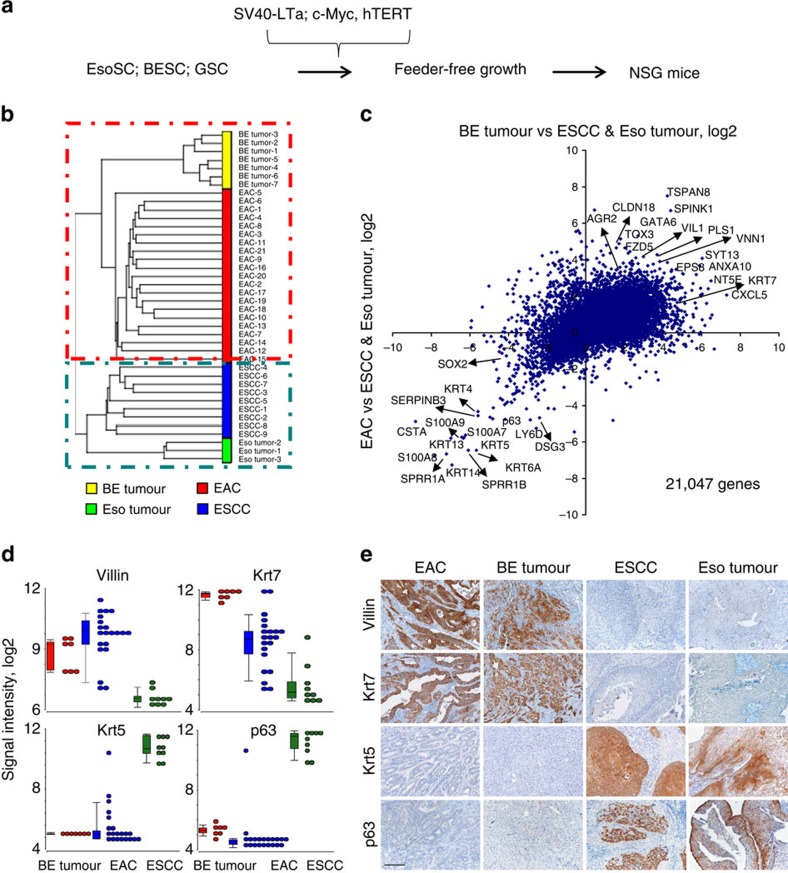
Tumours derived from transformed BESC and EsoSC pedigrees. (**a**) Schematic of stem cell transformation and xenografting in immunodeficient mice. (**b**) Dendritic relationship of gene expression profiles of EACs, ESCC and tumours developed in immunodeficient xenografted transformed Barrett's (BE tumour; *n*=7) and oesophageal (Eso tumour; *n*=3) stem cell pedigrees. (**c**) Correlation cluster analysis of genes expressed in BESC-derived tumours and those expressed in EAC. (**d**) Box plot of gene expression intensity from arrays for four genes marking tumours arising from xenografted transformed BESCs and EAC and squamous cell carcinomas. The top whisker shows maximum, the boxed zone shows 1st quartile, mediam and 3rd quartile, while the lower whisker shows minimum with outliers indicated (see Methods section). (**e**) Immunohistochemistry on sections of EAC, tumours derived from transformed BESC pedigrees (BE tumour), ESCC and tumours from transformed oesophageal stem cell pedigrees (Eso tumour). Scale bar, 7 mm.

The oesophagus is the site of origin of two major and very different cancers labelled squamous cell carcinoma and intestinal adenocarcinoma^26^. To address the question of cell-of-origin for these oesophageal cancers, we transformed Barrett's and oesophageal stem cell pedigrees *in vitro* using identical protocols^27^ involving retrovirally transduced SV40 large T antigen, c-Myc and hTERT and then injected them into NSG (*NOD.Cg-Prkdc*^*scid*^ *Il2rg*^*tm1Wjl*^*/SzJ*) mice^28^ (Fig. 4a). Transcriptome expression analysis of seven tumours originating from transformed Barrett's stem cell pedigrees and three tumours from transformed oesophageal stem cell pedigrees were compared by correlation cluster analysis with 21 EACs and 9 oesophageal squamous cell carcinomas (ESCC) already present in public NCBI databases^20^. This and related analyses segregated tumours from Barrett's stem cells with EACs and those from oesophageal stem cells with ESCCs (Fig. 4b,c and Supplementary Fig. 6a,b). We selected four genes highlighted by this analysis and compared their expression in the stem cell-derived tumour types along with ESCCs and adenocarcinomas. Expression was compared by signal intensity in microarrays and staining of histological sections of these tumours using antibodies to p63, Krt5, Krt7 and Vil1 (Fig. 4d,e and Supplementary Fig. 6c). We should add here that the mutational patterns in the tumours derived from *in vitro*-transformed Barrett's stem cells appeared much less chaotic than seen in EAC. For instance, while EAC presents at the genomic level as a tumour with some of the highest levels of chromosomal aberrations including copy number variation (CNV) and single nucleotide variation (SNV), those arising in immunodeficient mice from cells transformed *in vitro* with SV40 large T antigen, c-Myc and hTERT showed considerably fewer changes (Supplementary Fig. 7). We think this is likely due to the fact that the *in vitro* transformation protocol obviates the need for significant aneuploidy and rearrangements normally accompanying the evolution of malignancy. We also xenografted transformed gastric stomach stem cells in the same manner, and tumours from these are distinct from those from Barrett's stem cells and oesophagus stem cells (Supplementary Fig. 6d,e). Together these data support the concept that the two major forms of oesophageal cancer in fact arise from two very distinct stem cells that are otherwise committed to oesophageal epithelium and Barrett's intestinal metaplasia, respectively.

### Mutational analysis of Barrett's stem cells from 12 cases

**Figure 5:**
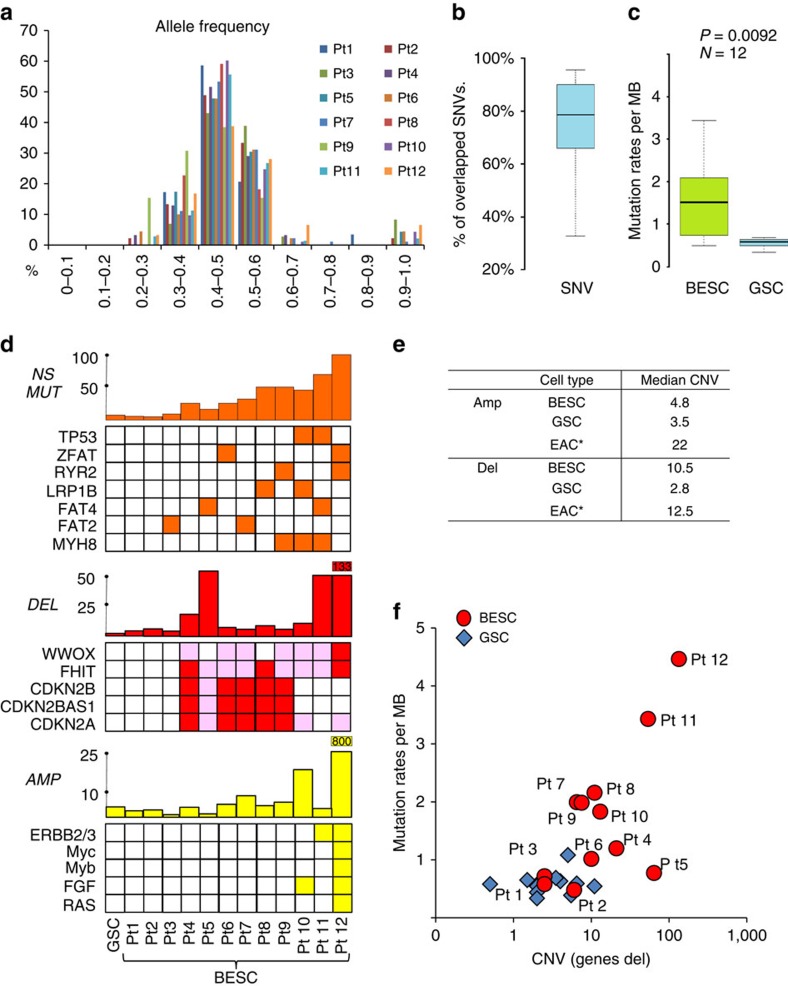
SNV in Barrett's stem cell pedigrees. (**a**) Histogram of somatic SNV allele frequency determined from exome sequencing of Barrett's stem cell pedigrees across the patient study group. (**b**) Distribution and median per cent overlap among SNVs determined from two independent BESC pedigrees (see Methods section). (**c**) Comparison of distribution of somatic mutation rates between BESC and GSC across the patient study group (see Methods section). (**d**) Summary of non-synonymous mutations (NS MUT), interstitial deletions (DEL) and interstitial amplifications (AMP) in Barrett's stem cells cloned from 12 cases without evidence of high-grade dysplasia. Histograms depict total number of events, whereas specific genes were listed beneath each histogram to highlight genes frequently altered in oesophageal and gastric adenocarcinoma^28^,^29^,^37^. (**e**) List of average number of CNV events as amplifications (Amp) or deletions (Del) in Barrett's and gastric stem cells as well as EAC^34^. (**f**) Graph depicting the distribution of BESC and GSC by mutation rate (SNV per Mb) and CNV (interstitial deletions)^50^,^51^.

Exome sequencing of Barrett's stem cell pedigrees revealed that allele frequencies of point mutations clustered around 0.4–0.5 as expected for clonal populations (Fig. 5a). These allele frequencies underscore the robustness of genomic analysis on stem cell pedigrees as most cells in a typical Barrett's biopsy are of stroma and ‘squamous islands'^6^ and therefore obscure genomic data on Barrett's cells. We found a high degree of concordance in the SNV of the two independent pedigrees of the Barrett's stem cells, suggesting that at least 80% of the SNVs we discovered were present in the Barrett's lesion before *in vitro* cultivation (Fig. 5b). In general, the Barrett's stem cell pedigrees also had both a higher rate of SNVs than their patient-matched stomach stem cells and a much broader distribution of these rates across the 12 patients (Fig. 5b and Supplementary Data 2). While patients with ‘suspected low-grade dysplasia' (Patients 11, 12) had the highest mutation rates, we also saw examples of high rates of mutations in both long (patients 8 and 9)- and short (patient 7 and 10)-segment Barrett's (Fig. 5c and Supplementary Data 3). Many of the nearly 100 genes that sustained non-synonymous mutations in the Barrett's stem cell pedigrees are the same as those reported to appear in multiple cases of EAC^29^, and some of these (for example, *FAT2*, *FAT4*, *MYH8*, *RYR2*, *TP53 and ZFAT*) showed up in a recurrent manner even in the relatively small cohort of patients examined here (Fig. 5d, Supplementary Figure 8 and Supplementary Table 2).

We also assessed CNV in these cases using high-density single nucleotide polymorphism (SNP) arrays on two independent pedigrees of Barrett's and stomach stem cells from each patient as well as on venous blood. The range of CNV events in the Barrett's pedigrees, including deletions and amplifications, varied widely from patient to patient with as few as three events involving no genes to nearly one thousand events affecting several thousand genes (Fig. 5d, Supplementary Table 3 and Supplementary Data 4). In general, these CNV events were in the form of relatively small interstitial deletions and amplifications rather than chromosomal aneuploidies. Approximately half of the patients had CNV deletions in loci affecting *FHIT*, *CDKN2A and WWOX* reported to be common in both Barrett's and EAC^30^,^31^,^32^,^33^ (Fig. 5d and Supplementary Table 4). These common loci were recurrent in 50–75% of the patient's Barrett's stem cells but never in the patient-matched stomach stem cells (Supplementary Data 4). More than a third of the Barrett's stem cells (patients 4, 6, 7, 8 and 9) sustained homozygous losses of *CDKN2A*, *CDKN2b*, *CDKN2B-AS1 and MTAP*, along with a lower frequency loss of both alleles of FHIT (Fig. 5d), favouring the notion that these losses are providing selective advantage to these cells^34^. Significantly, the median number of CNV deletions we found in these 12 Barrett's cases (10.5) was much higher than found in patient-matched stomach (2.8) but nearly the same as the number of CNV deletions seen in advanced EAC^35^ (Fig. 5e). In contrast, CNV amplifications were not common in either Barrett's or patient-matched stomach stem cells and certainly far fewer than seen in EAC (Fig. 5d,e and Supplementary Table 4). Thus while the deletions we observed in Barrett's stem cells appeared very similar in numbers and even the genes affected to those seen in EAC^35^,^36^ (Fig. 5e), Barrett's stem cells generally lacked the p53 mutations and interstitial amplifications of receptor tyrosine kinases and other established oncogenes found in these cancers (Fig. 5d). However, patients 10, 11 and 12, the latter two of which were denoted in pathology workups as suspect for low-grade dysplasia, run counter to this generalization with either p53 mutations or extensive amplifications of loci including *ERBB2*, *FGFR1*, *GATA4*, *GATA6*, *KLF5*, *KRAS*, *MYC*, *SOX9 and VEGFA* seen in EAC^29^,^30^,^31^,^32^,^33^,^37^ (Fig. 5d). Overall the Barrett's stem cells of the cases examined here showed broad distribution of both point mutations and structural variation that suggested their genomic instability compared with the stomach stem cells from the same cohort (Fig. 5f). These data lend support to the notion that deletions accompany or favour the evolution of Barrett's whereas amplifications in particular, along with p53 mutations, drive its conversion to dysplasia and adenocarcinoma.

## Discussion

The pre-emptive eradication of precursor lesions would ideally target a hypothetical ‘stem cell' responsible for their regenerative growth much as efforts to target ‘cancer stem cells' would be for controlling frank carcinomas. The present work uses technology recently developed for growing ‘ground state' intestinal stem cells^16^ to identify stem cells of Barrett's oesophagus, the precursor of EAC, from a cohort of Barrett's cases. The cellular, gene expression and genomic analyses of the stem cells provide insights into Barrett's as a unique biological entity in the distal oesophagus, the role of mutations in the origin and progression of Barrett's, and finally the feasibility of specifically targeting Barrett's stem cells as a means of precluding lethal EAC.

Barrett's intestinal metaplasia is a regenerative epithelia that is histologically distinct from adjacent oesophageal and gastric epithelia^6^. Consistently, the particular stem cell cloning technology we developed for intestinal stem cells enabled the isolation of distinct oesophageal, Barrett's and stomach stem cells from selected biopsies at the distal oesophagus of 12 Barrett's patients. In particular, the approach of sampling discrete colonies arising from single cells proved essential for separating Barrett's stem cells from those of ‘squamous islands' that frequently co-mingle with Barrett's glands^38^. Using gene expression profiles of these defined pedigrees, we could readily distinguish the stem cells of Barrett's oesophagus from their counterparts of the squamous epithelium of the oesophagus and with a 131 gene signature from those of the more closely related gastric epithelia. The distinctions between Barrett's stem cells and those of gastric epithelia became even more evident following ALI differentiation, as goblet, endocrine and Paneth cells could be detected in Barrett's but not in stomach. This multipotency of the Barrett's stem cells, coupled with their long-term self-renewal capacity, supports the notion that these are *bona fide* stem cells and distinct from those of the oesophagus and stomach epithelium. Consistent with this concept, *in vitro*-tranformed Barrett's stem cells yield tumours in immunodeficient mice that are similar to those of EAC by both histology and gene expression, whereas transformed oesophageal stem cells exclusively yielded squamous cell carcinomas. These findings support the concept that these two very different oesophageal cancers indeed arise from stem cells of distinct lineages.

The variations in mutational spectra among the Barrett's cases reported here support our earlier supposition that Barrett's arises by opportunistic expansion rather than activating mutations^22^. The concept of mutation-free, opportunistic expansion of Barrett's was founded on the extremely rapid development of a Barrett's-like metaplasia in the p63 null mouse. This model would predict that Barrett's in humans also initiates without mutations. This is precisely what was observed in Barrett's stem cells from at least three patients examined in this present study (for example, patients 1, 2 and 3), which showed levels of somatic structural and sequence variation equal to or even less than that of nearby normal stomach epithelia. These findings are consistent with the notion that clinically defined Barrett's initially arises in the absence of established driver mutations.

Our genomics analyses also suggest that Barrett's stem cells of most patients analysed can accumulate a high degree of structural and sequence mutations that likely enhance, but in no way ensure, their potential for transformation. These alterations, in addition to deletions at fragile sites, include a range of heterozygous mutations in other genes observed in cancer and specifically in EAC^29^,^37^. As all the cases included in this study were Barrett's without high-grade dysplasia, population studies would predict that only 5–15% of these cases would advance to adenocarcinoma^1^. Thus it is probably fair to assume that most of the cases studied here, irrespective of their individual mutational profiles, would not progress to high-grade dysplasia and adenocarcinoma within the patients' lifetimes. Perhaps the most salient difference between the mutational profiles in the majority of Barrett's stem cells representing cases that might never progress (for example, cases 4, 5, 6, 7, 8 and 9) to frank dysplasia and adenocarcinoma is the absence of both p53 mutations and amplified loci harbouring receptor tyrosine kinases (*EGFR*, *c-MET*, *ERBB2*, *FGFR1*, *FGFR2*) and other oncogenes such as *KRAS*, *VEGFA*, *MDM2 and MYB*)^35^,^36^. In contrast, cases 10, 11 and 12 already display either p53 mutations or oncogene amplification typical of cancer, and thus would seem to be at higher risk for progression. These data, combined with our observation that *in vitro*-transformed Barrett's stem cells yield EAC, support the precursor role of Barrett's stem cells in this cancer as well as specific roles of p53 mutations and of amplified oncogenes in this conversion.

The present study underscores the utility of stem cell analysis and pedigree development for addressing cancer precursors and human disease in general. Barrett's itself presents as a complex network of intestinal metaplasia interspersed with normal oesophageal squamous islands, stromal cells, and perhaps gastric-like columnar cells that limits molecular analyses^6^,^39^. Organoids generated from Barrett's biopsies^40^ could include conceivably many of these contaminating cell types. However, stem cell pedigrees described herein resolve this complexity and can be propagated for multifaceted analyses that ultimately trace to a single cell. We anticipate the Barrett's-specific cell surface markers such as Cdh17 represent targets for monoclonal antibody drugs directed at these stem cells, and that the cloned stem cells themselves will help in assays for validating such drugs as well as the basis for selecting small molecule drugs directed at Barrett's. These stem cells are also likely to have roles in defining the precise genetic and epigenetic processes that accompany the evolution from precancerous lesion to lethal carcinoma. Consistent with the ‘clonal dominance' hypothesis for Barrett's evolution^41^, independent Barrett's pedigrees from the same patient share a core set of mutations in cancer-related genes as well as sets of unique mutations that may reflect stochastic events that underlie progression via such clonal dominance. We also anticipate that the strategies presented here will help in resolving the complexities of defining ‘driver' versus ‘passenger' mutations^42^ in the progression to cancer.

Finally, Barrett's oesophagus is an intestinal metaplasia not dissimilar to the gastric intestinal metaplasia implicated in gastric adenocarcinoma^9^ and perhaps to other metaplastic precursor lesions that precede certain pancreatic and bladder cancers. As a group these metaplastic precursors give rise to some of the most aggressive and poorly responding human cancers. If the observations presented here for Barrett's oesophagus prove to be generalized across these lesions, the range of pathways for the initiation of precursor lesions^43^ needs to be expanded and exploited in strategies for preventing cancers arising from metaplastic precursors.

## Methods

### Stem cell derivation from endoscopic biopsies

Patient's with Barrett's were recruited for this study under informed consent consistent with institutional review board protocols of the National Hospital Group Domain-Specific Review Board Approval #2010/00700 of the Ministry of Health, Singapore or of the Maine Medical Center (FO 301A). All biopsies were derived from patients either at their initial endoscopy or within 1 year of initial diagnosis during routine follow-up endoscopy. Endoscopic biopsies (1 mm) of the distal oesophagus of patients with Barrett's oesophagus were collected into RPMI media (Gibco) with 2% fetal bovine serum and subsequently digested in 2 mg ml^−1^ collagenase A (Roche) at 37 °C for 1.5 h. Cells were washed by centrifugation in RPMI, digested with 0.5% trypsin (Gibco) 10 min, passed through a 40-um Nylon mesh (Falcon), and seeded onto a feeder layer of lethally irradiated 3T3-J2 cells in c-FAD media^17^ modified to SCM-68 media^16^ by the addition of 125 ng ml^−1^ R-spondin1 (R&D systems, USA), 1 μM Jagged-1 (AnaSpec Inc, USA), 100 ng ml^−1^ human Noggin (Peprotech, USA), 2.5 μM Rock-inhibitor (Calbiochem, USA), 2 μM SB431542 (Cayman chemical, USA) and 10 mM nicotinamide (Sigma-Aldrich, USA). The culture medium was changed every 2 days. Cells were passaged every 7–10 days following treatment with 0.25% trypsin to generate single-cell suspensions. For pedigree generation, well-separated colonies were isolated by cloning rings, digested with trypsin, and the resulting single-cell suspension filtered through a 40-um Nylon mesh (Falcon) cell strainer and plated onto 3T3-J2 feeder cells (Rheinwald and Green, 1975). All gene expression and genomic analyses were performed on cells derived from passage 5 (P5) cultures or earlier passages.

### Histology and immunostaining

Histology, immunohistochemistry and immunofluorescence were performed using standard techniques, processed at the Histology Core at the Institute of Molecular and Cellular Biology at A*STAR and imaged at the Institute of Medical Biology, A*STAR or at the Jackson Laboratory for Genomic Medicine. Immunofluorescence staining was performed on 4% paraformaldehyde-fixed, paraffin-embedded sections or frozen sections. Antibodies used in this study are listed in Supplementary Table 5. All images for section slides were captured by using Axio Observer.Z1 fluorescence microscope (Zeiss) with monochrome MR Rev3 and colour ICc1 (Zeiss) cameras and Axiovision 4.8 software (Zeiss) or LSM 510 confocal microscope (Zeiss) with LSM software. Bright field cell culture images were obtained on an Eclipse TS100 microscope (Nikon) with Digital Sight DSFi1camera (Nikon) and NIS-Elements F3.0 software (Nikon).

### Stem cell differentiation

ALI culture of single-cell-derived pedigrees of epithelial stem cells was performed as described^18^. For differentiation, immature stem cell pedigrees were digested by 0.05% trypsin for 30–60 s. The plate was shaken manually to remove the feeders. Stem cell clones were removed by pipetting up and down several times. The cluster of stem cell clones were neutralized and plated onto Transwell (Corning) membranes, grown to confluences, and exposed to an ALI for 10 days during which media was changed every third day. After 10 days, the differentiated structures were collected for sectioning, immunohistochemistry and immunofluorescence staining and RNA collection.

### Xenografts of transformed stem cells

Barrett's, stomach and oesophageal stem cells were transformed by transduction of retroviruses expressing c-Myc, hTERT and SV40 large T antigen as described^27^. In brief, 200,000 stem cells were plated onto a lawn of feeder cells in 3 cm culture dishes and transduced 3 days later. After 48 h, cells were split 1:5 onto new lawns and grown and passaged for 4 weeks before plating onto culture plates without feeder cells for an additional 4 weeks. Individual colonies were selected and tested for growth in soft agar, and positive colonies selected for expansion and transplantation. One million transformed stem cells were injected subcutaneously into five 6-week-old male NSG (*NOD.Cg-Prkdc*^*scid*^ *Il2rg*^*tm1Wjl*^*/SzJ*) mice^28^ in protocols approved under BRC IACUC #110643 at the Agency for Science Technology and Research (A*STAR) Singapore and the Genome Institute of Singapore, Singapore. Visible tumours appeared typically at 2 months and were collected following euthanasia and analysed by histology and expression microarray.

### RNA and DNA sample preparation

For stem cell colonies, RNA was isolated using the PicoPure RNA Isolation Kit (Life Technologies, NY, USA). For xenografted tumours from transformed Barrett's, stomach and oesophageal stem cells, RNA was isolated with the TRIZOL and PicoPure RNA Isolation Kit (Life Technologies) and purified with RNeasy Mini kit (Qiagen, CA, USA). RNA quality (RNA integrity number (RIN)) was measured by analysis Agilent 2100 Bioanalyzer (Agilent Technologies, CA, USA). RNAs having a RIN >8 were used for microarray analysis. Genomic DNA was extracted with DNeasy Blood & Tissue kit (Qiagen) from patient-matched Barrett's and stomach stem cells and from blood for CNV analysis and exome sequencing.

### Expression microarray

RNAs obtained from immature colonies and differentiated cells on Matrigel and ALI culture were amplified using the WT Pico RNA Amplification System V2 and Encore Biotin Module (NuGEN Technologies, CA, USA). All samples were prepared according to manufacturer's instructions and hybridized onto GeneChip Human Exon 1.0 ST Array (Affymetrix, CA, USA). For xenografted tumours, extracted RNAs were amplified with 3' IVT Express Kit (Affymetrix). Amplified RNAs were hybridized onto GeneChip Human U133 plus 2.0 Arrays (Affymetrix). GeneChip operating software was used to process all the Cel files and calculate probe intensity values. To validate sample quality, quality check was conducted using Affymetrix Expression Console software. The intensity values were log2 transformed and imported into the Partek Genomics Suite 6.6 (Partek Incorporated, MO, USA). Exons were summarized to genes and a one-way ANOVA was performed to identify differentially expressed genes. *P* values and fold-change numbers were calculated for each analysis.

### Bioinformatics for gene expression

Unsupervised clustering and Heatmap generation were performed with sorted data sets by Euclidean distance based on average linkage clustering and PCA was conducted using all or selected probe sets by Partek Genomics Suite 6.6. Gene set enrichment analysis^44^ was performed to compare undifferentiated and differentiated Barrett's stem cells. Box plots and a scatter plot were created with normalized signal intensity of genes exported by Partek Genomics Suite 6.6. Affymetrix U133 plus 2.0 array data of Barrett's oesophagus and normal oesophageal epithelium (GSE13083)^19^, EAC and ESCC (GSE26886)^20^ and Human Exon 1.0 ST array of stomach biopsy (GSE34619) were downloaded from GEO data sets for heatmap, unsupervised clustering, box plot and scatter plot generation by Partek Genomics Suite 6.6 and Microsoft Excel. All the microarray data in this study are publically available (GSE64894, GSE65013 and GSE49292).

### Copy number variation

For CNV analysis of stem cell pedigrees and patient-matched blood, genomic DNA samples were genotyped with Illumina HumanOmniZhonghua BeadChip Kit (Illumina, CA, USA) following the manufacturer's instructions. All data have been submitted as GEO ID: GSE68664.

Analysis of BeadChip was performed using GenomeStudio Software (Illumina). Illumina high-density SNP genotyping data was converted to kilobase-resolution detection of CNV. CNV detected in patient-matched blood samples are considered as germline CNVs and removed in the analysis. The data was generated by Partek Genomics Suite 6.6 and PennCNV. The box plot was created by BoxplotR (http://boxplot.tyerslab.com/).

### Exome sequencing

For Exome sequencing, five micrograms of genomic DNA per sample were sheared using a Covaris S1 Ultrasonicator (Covaris, MA, USA). All data has been submitted under SRA ID: SRP058410.

Sheared genomic DNA was end-repaired, A-tailed and Adaptor-ligated. Exon capture was performed using a SeqCap EZ Human Exome Library (Roche Nimblegen, WI, USA) and SureSelect Human All Exon V4 (Agilent, DE, USA). Multiplexed libraries were sequenced on an Illumina HiSeq sequencer using 101 bp paired-end reads. Reads were uniquely mapped to the reference genome (UCSC hg19) using Burrows-Wheeler Aligner^45^. PCR duplicates were removed using PICARD-1.48 (http://picard.sourceforge.net). Genome Analysis Toolkit (GATK-1.0.5974) (ref. 46) was used to realign reads near indels and to recalibrate base quality values. Potential contaminant reads were detected by alignment to the mouse genome (UCSC mm9) and those containing <3 mismatches were removed from further analysis. SNVs were called in each sample separately using SAMtools v0.1.1 (ref. 47), (coverage threshold=10, SNP-quality threshold=40 and Consensus-quality threshold=30) and LoFreq (ref. 48) in the exon-targeted regions. Identical variant calls in Barrett's and stomach when compared with matched blood samples were used to identify germline SNVs. Barrett's- and stomach-specific SNVs were identified by filtering for common variants (with blood) seen in SAMtools or LoFreq calls and testing for sufficient coverage using LoFreq^49^,^50^. SIFT^51^ was used to annotate and assess the impact of non-synonymous SNVs. Sanger sequencing validation was performed using primers designed with Primer3 software version 4.0 (http://frodo.wi.mit.edu/). Extracted genomic DNA was amplified with Titanium taq polymerase (Clontech Laboratories, CA, USA) and purified PCR products were sequenced in the forward directions using ABI PRISM BigDye Terminator Cycle Sequencing Ready Reaction kits and an ABI PRISM 3730 Genetic Analyzer (Applied Biosystems, CA, USA). The Box plots were prepared by BoxplotR (http://boxplot.tyerslab.com/).

## Additional information

**How to cite this article**: Yamamoto, Y. *et al.* Mutational spectrum of Barrett's stem cells suggests paths to initiation of a precancerous lesion. *Nat. Commun.* 7:10380 doi: 10.1038/ncomms10380 (2016).

## Supplementary Material

Supplementary Information Supplementary Figures 1-8 and Supplementary Tables 1-5

Supplementary Data 1 BESC and GSC gene signature (131 genes, 2.5 fold and p < 0.001)

Supplementary Data 2 Summary of nonsynonymous and synonymous SNV across 12 patients

Supplementary Data 3 Exone sequencing run analysis for BESCs and GSCs from Barrett's patients

Supplementary Data 4 List of CNV in BESC and GSC across 12 patients
