# Supplementary material for: Mutational spectrum of Barrett's stem cells suggests paths to initiation of a precancerous lesion
Source: Nat Commun. 2016 Jan 19;7:10380. doi: 10.1038/ncomms10380 (PMC4735693; doi:10.1038/ncomms10380)
Supplement: Supplementary Information — Supplementary Figures 1-8 and Supplementary Tables 1-5 [file ncomms10380-s1.pdf]

Supplementary Fig. 1

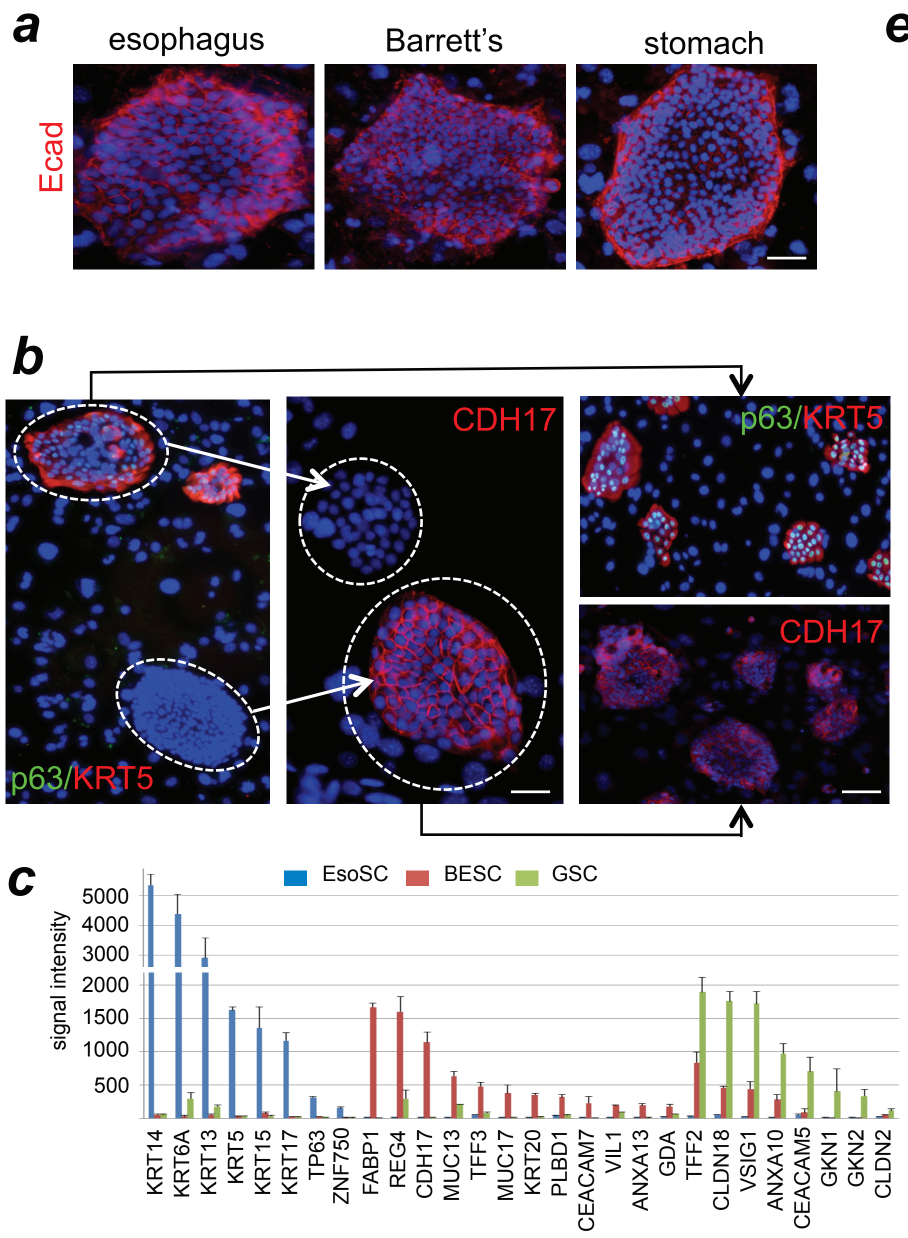

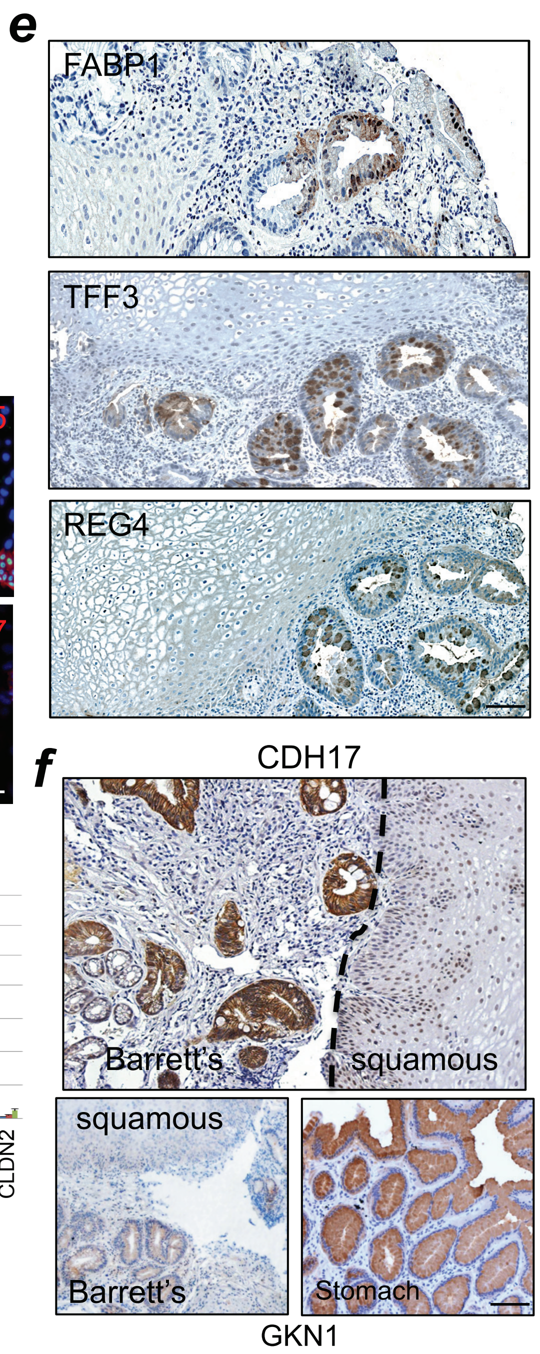

d

	SOX2	SOX9	GKN1	CDH17	p63
EsoSC	100%	0%	0%	0%	100%
BESC	0%	100%	0%	100%	0%
GSC	100%	100%	100%	0%	0%

Supplementary Fig. 1. Cloning Barrett's stem cells

a. Representative immunofluorescence labeling of colonies of EsoSC, BESC, and GSC with antibodies to the epithelial marker E-cadherin (red) with nuclei counterstained with DAPI (blue); Scale bar, 100um. n = 12 biological replicates. **b.** Markers for Barrett's (Cdh17) and esophageal squamous stem cells (p63/Krt5) distinguish colonies from Barrett's biopsies that include both tissues (*left*). DNA staining by DAPI (blue). Subsequent colony sampling and replating to generate pure Pedigrees with uniform marker profile (*middle, right panels*). Scale bar, 200um. n = 12 biological replicates. **c.** Relative expression of genes in the indicated stem cells associated with esophageal squamous, Barrett's, and stomach epithelia. Error bars indicate standard deviation (SD) from mean (biological triplicate).
d. Percentage of differential immunophenotype of stem cell colonies (n>10) derived from respective pedigrees.
e. Immunohistological staining of Barrett's stem cell markers in biopsies of Barrett's esophagus. Scale bar, 100um.
f. Cdh17 antibody immunohistochemistry staining (brown) at the neojunction between Barrett's and the esophageal squamous epithelium. *below,* Immunohistochemistry of anti-gastrokine 1 (Gkn1) antibodies in biopsies of Barrett's and stomach epithelium. Scale bar, 200um.

Supplementary Fig. 2

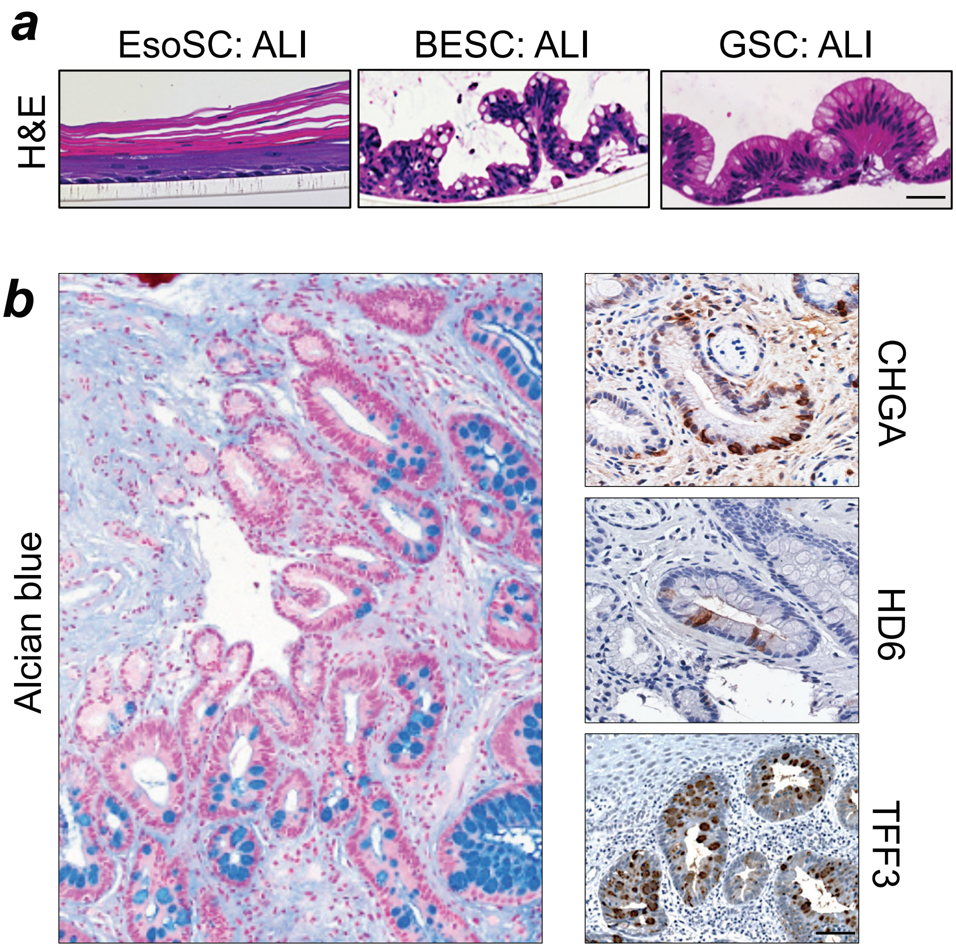

Supplementary Fig. 2. Properties of Barrett's stem cells
a. Differentiation of esophageal (EsoSC), Barrett's (BESC), and stomach (GSC) stem cell pedigrees in 3-D air-liquid interface (ALI) cultures revealed by histological sections of epithelia stained by H&E. Scale bar, 100um. n = 12 biological replicates. **b.** Staining of Barrett's biopsy sections with Alcian blue (left) and immunohistochemistry with antibodies to CHGA, HD6, and TFF3 (right panels). Scale bar, 100um. n = 12 biological replicates.

Supplementary Fig. 3

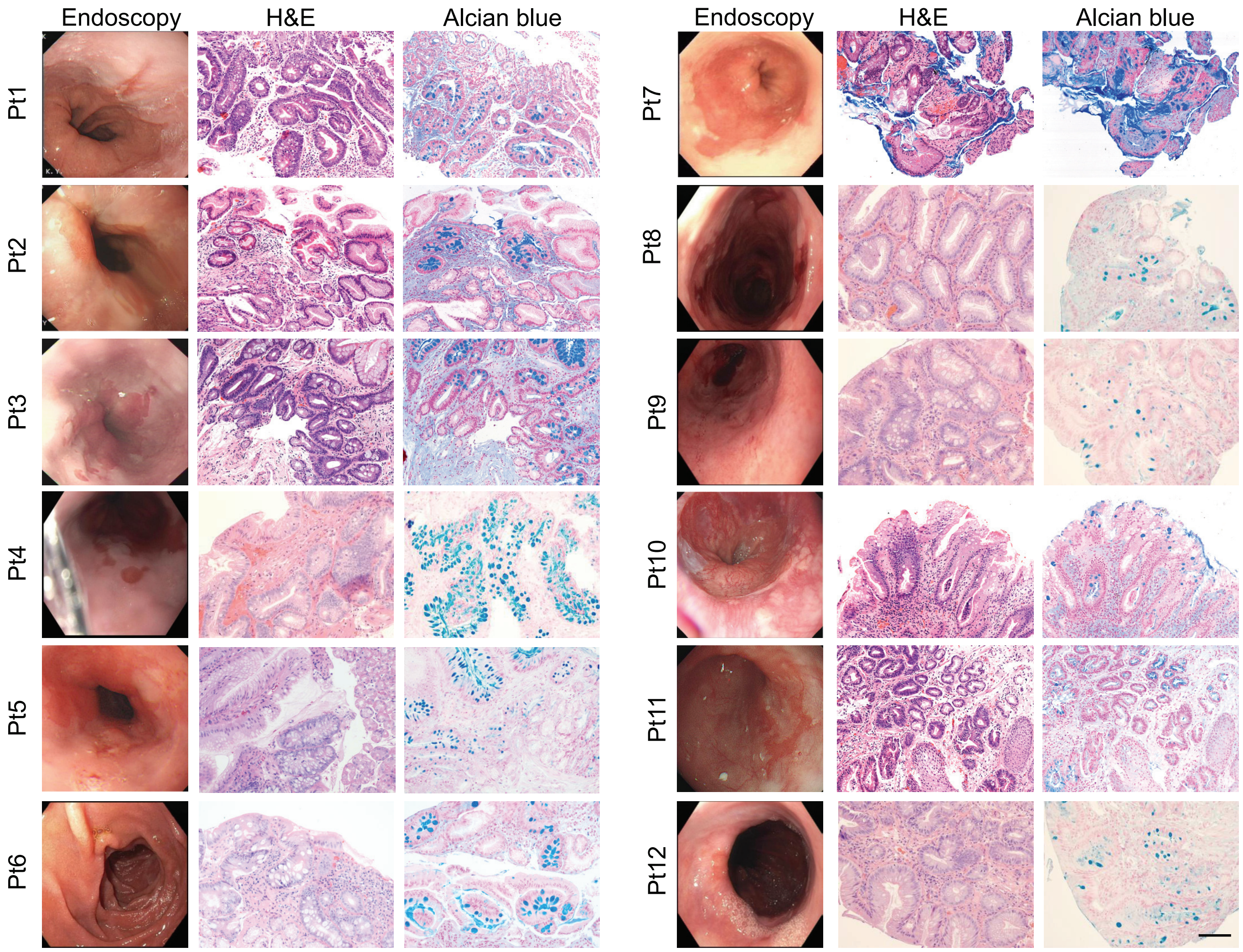

Supplementary Fig. 3. Endoscopic imaging and histology of 12 BE patients.
Endoscopic imaging of distal esophagus (left); H&E (middle), and Alcian blue staining (right) of biopsies of distal esophagus in study group cases. Scale bar, 200um.

Supplementary Fig. 4

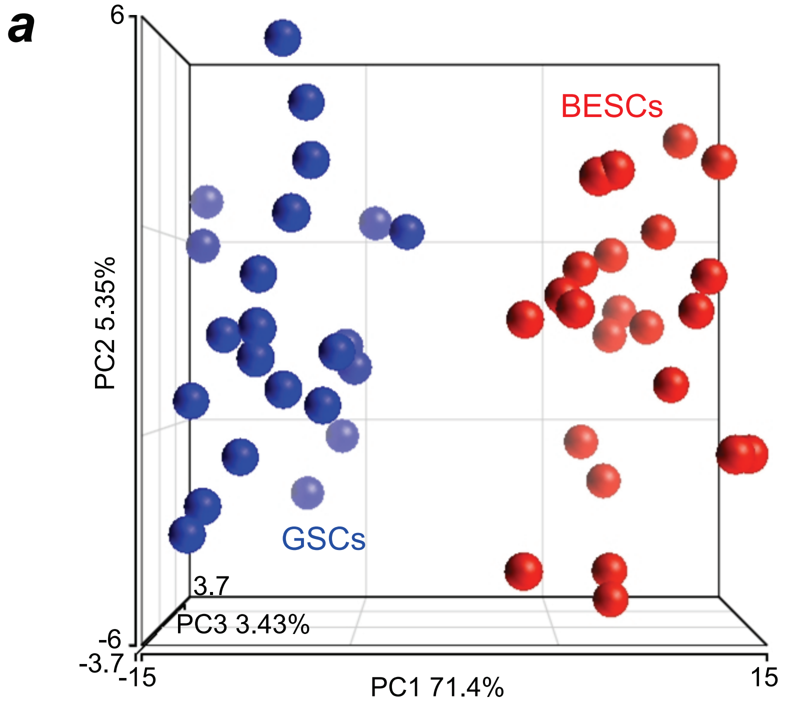

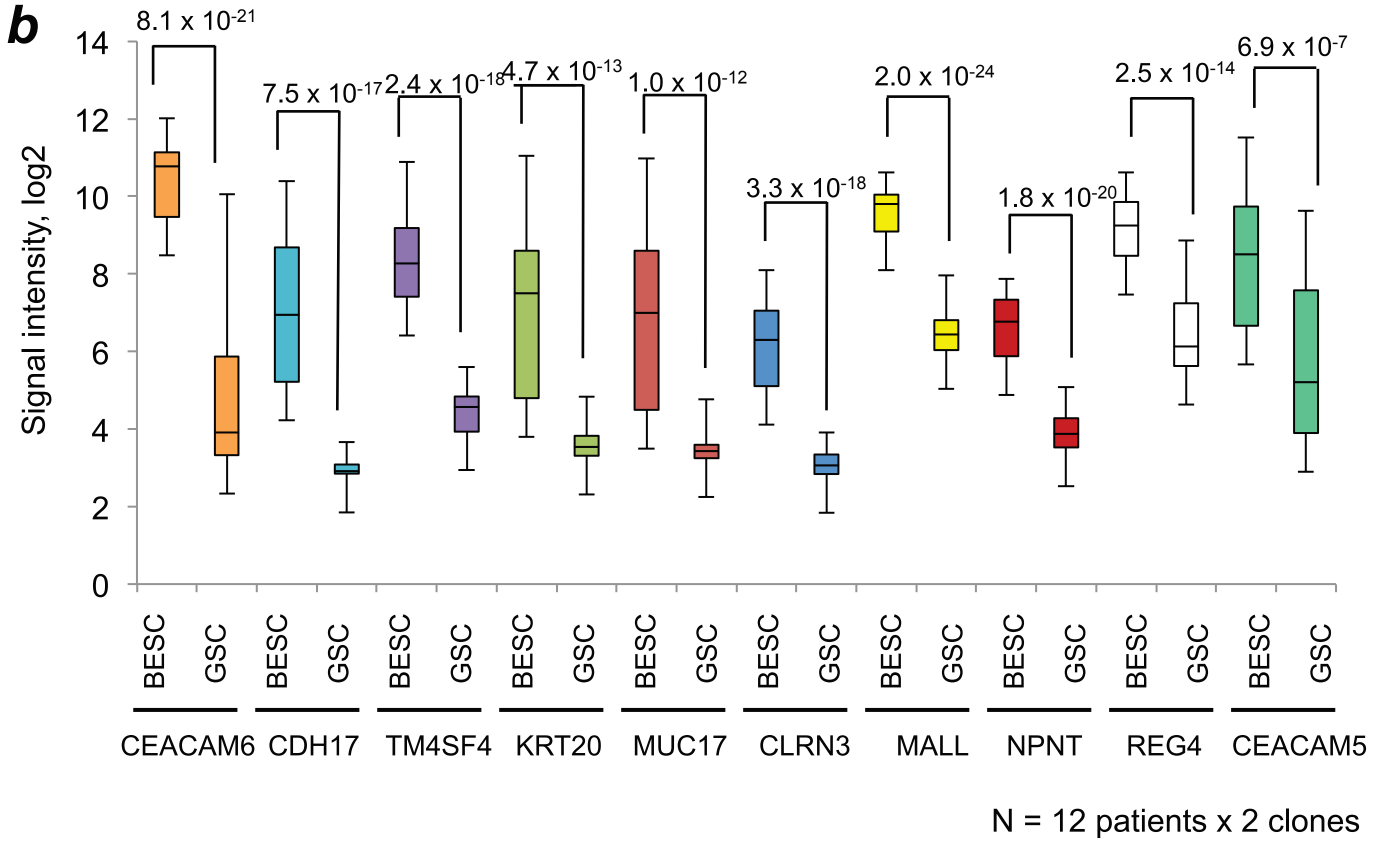

Supplementary Fig. 4. Whole transcriptome analysis of BESC and GSC in 12 patients
a. PCA mapping of whole genome expression data of BESC and GSC in 12 patients (each patient: 2 independent clones). **b.** Box plots of top 10 highly expressed genes in BESC as compared with GSC. p values were calculated by student's t-test.

Supplementary Fig. 5

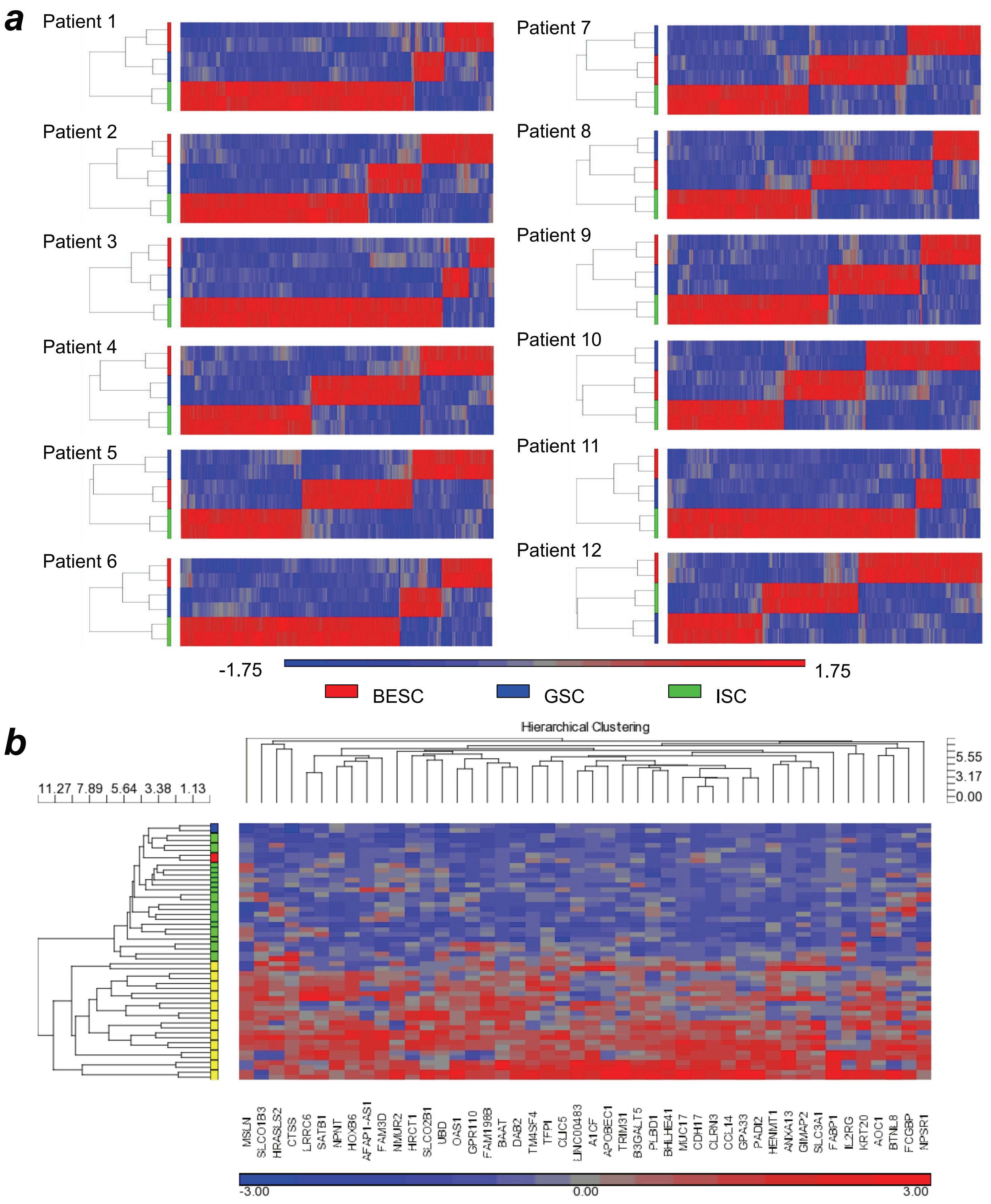

Supplementary Fig. 5. Gene expression comparison of stem cells
a. Heatmap of differential gene expression in BESC, GSC, and ISC among the 12 Barrett's cases. **b.** Barrett's stem cell-specific gene expression when compared against stomach and intestinal stem cells across the 12 Barrett's cases.

Supplementary Fig. 6

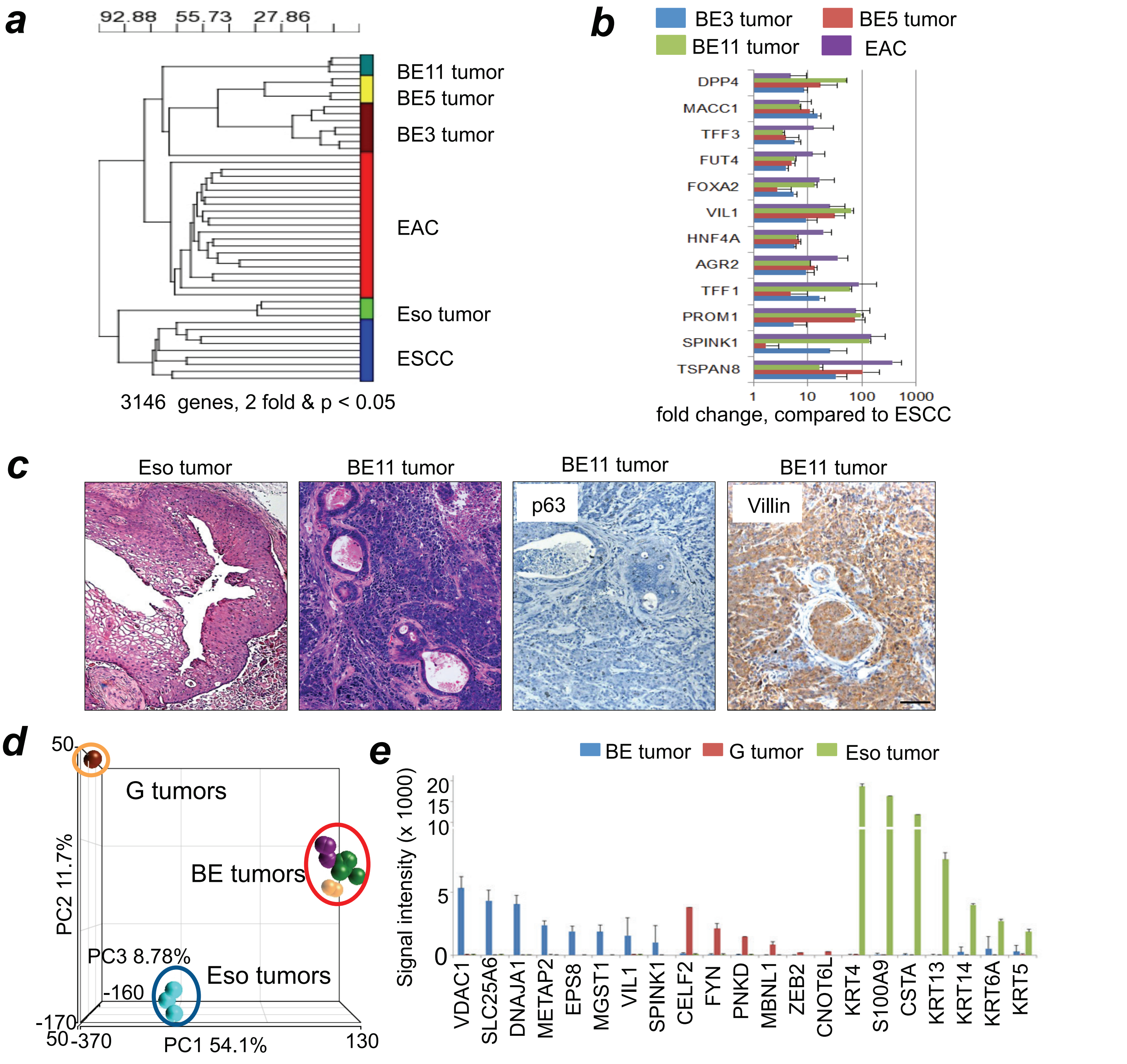

Supplementary Fig. 6. Tumors from transformed Barrett's, stomach, and esophageal pedigrees
a. Unsupervised clustering of gene expression data from tumors derived from transformed Barrett's stem cells of Patient 1 (BE1), Patient 4 (BE4), Patient 11 (BE11) and transformed EsoSC xenografted in mice with those of esophageal adenocarcinomas (EAC) and esophageal squamous cell carcinomas (ESCC). Clustering was conducted with 3146 genes (EAC vs. ESCC, 2-fold and p-value < 0.05) (GSE26886). **b.** Esophageal adenocarcinoma marker expression in BE1 tumors (n=7), BE4 tumors (n = 4), BE11 tumors (n=3) and EAC. Fold expression changes of BE1 tumor, BE4 tumor, BE 11 tumor and EAC relative to ESCC expression are shown in the graph. Error bars depict SD of the mean. **c.** Histological staining and immunohistochemistry on sections of tumors derived from transformed esophageal stem cells (tEsoSC) and those from transformed Barrett's stem cells (tBESC). Staining with antibodies to p63 and villin are shown. Scale bar, 200um. n = 3 biological replicates. **d.** PCA map of transformed BESC, GSC and EsoSC with using whole transcriptome data. **e.** Differentially expressed genes among transformed BESC, GSC and EsoSC.

Supplementary Fig. 7

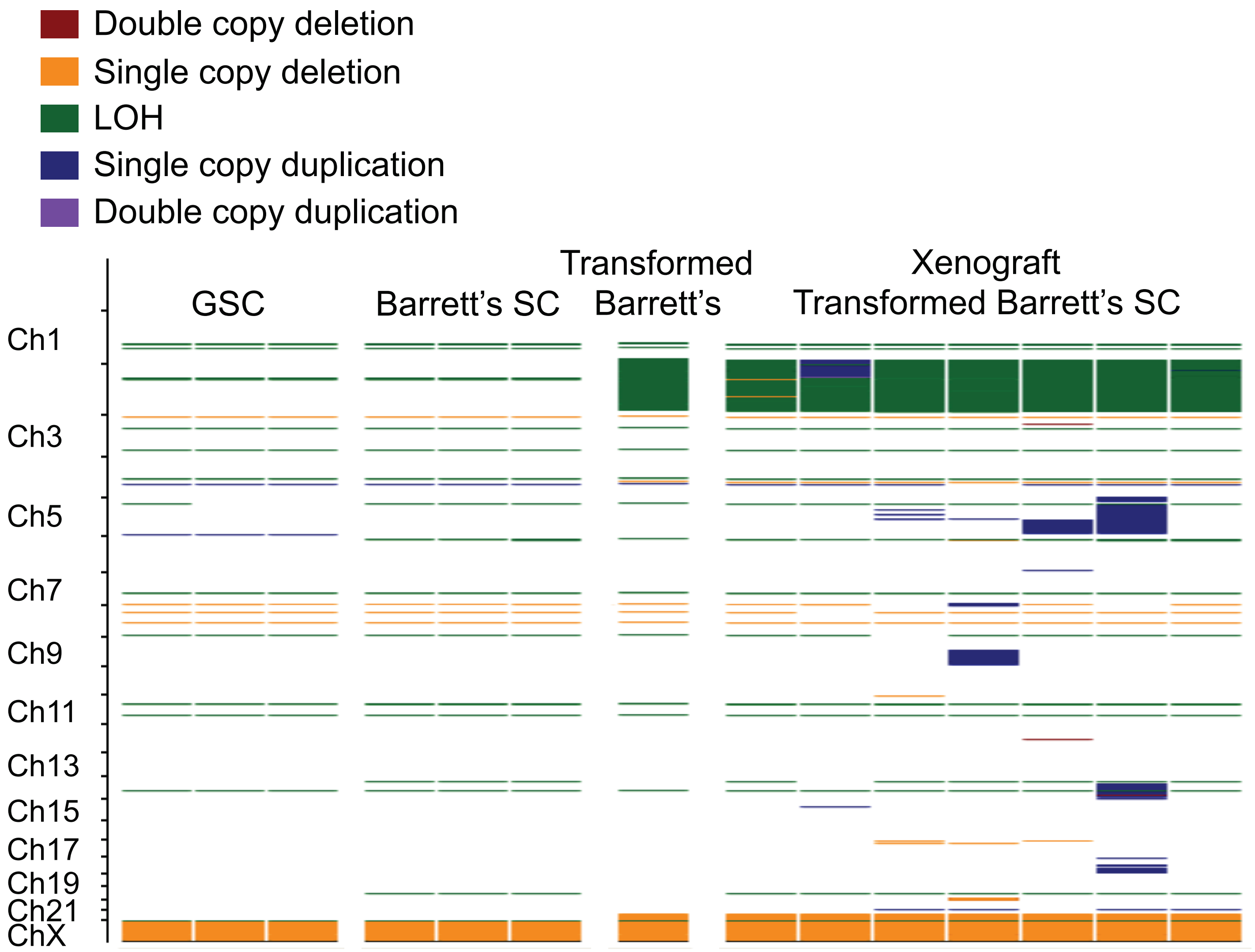

Supplementary Fig. 7. Copy number variation in tumors from transformed Barrett's stem cells
Summary of copy number variation from human genome in stomach stem cells (GSC), Barrett's stem cells before and after *in vitro* transformation, and in the tumors that arose in each of seven immunodeficient mice 6-8 weeks after inoculation of one million transformed cells.

Supplementary Fig. 8

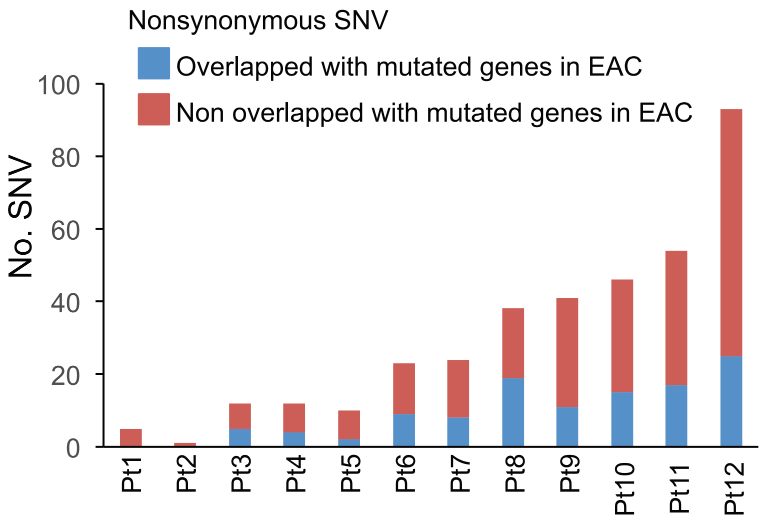

Supplementary Fig. 8. SNVs in BESC and esophageal adenocarcinoma
Histogram of nonsynonymous mutations in BESC that overlap (blue) or are unique (red) compared with those seen in esophageal adenocarcinoma.

Supplementary Table 1

Cohort Information

Sample	Code	Age	Gender	Ethnicity	Status	Dysplasia
patient 1	P63009	47	M	others	Short segment	No
patient 2	P63019	56	M	Chinese	Short segment	No
patient 3	P63001	67	M	Chinese	Short segment	No
patient 4	023BS	54	F	Caucasian	Short segment	No
patient 5	P63020	50	F	Chinese	Short segment	No
patient 6	005GS	71	M	Caucasian	Short segment	No
patient 7	P63008	61	M	Indian	Short segment	No
patient 8	010JD	59	M	Caucasian	Long segment	No
patient 9	020MG	69	F	Caucasian	Long segment	No
patient 10	P63038	42	M	Chinese	Short segment	No
patient 11	P63007	73	M	Chinese	Short segment	low grade
patient 12	007RC	81	M	Caucasian	Short segment	low grade

Supplementary Table 1. Characteristics of 12 Barrett's cases in study

Supplementary Table 2

Gene	Mutation type	RefSeq	Nucleotide postion and change	Amino acid change	Sample ID
MYH8	NM_002472	Missense	chr17,10307717,C/T	R873Q	9
MYH8	NM_002472	Missense	chr17,10304701,T/G	K1000T	10
MYH8	NM_002472	Missense	chr17,10315748,G/A	T452I	11
CSMD3	NM_198123	Missense	chr8,113267566,T/G	N3318T	8
CSMD3	NM_198123	Missense	chr8,113933896,T/G, 113569123,T/C	E531D, D1368G	10
FAT2	NM_001447	Missense	chr5,150908789,A/G	Y3326H	3
FAT2	NM_001447	Missense	chr5,150931062,T/A	E1421V	7
FAT4	NM_001291285	Missense	chr4,126400925,G/A	R4168H	5
FAT4	NM_001291285	Missense	chr4,126372174,T/C	Y1633H	11
LRP1B	NM_018557	Missense	chr2,141819690,A/G	L389P	8
LRP1B	NM_018557	Missense	chr2,141533692,T/A	K1825N	10
RYR2	NM_001035	Missense	chr1,237664064,G/T	D753Y	9
RYR2	NM_001035	Missense	chr1,237947124,G/A	D4038N	12
TP53	NM_001126117	Missense	chr17,7578265,A/G	I195T	10
TP53	NM_001126117	Missense	chr17,7577550,C/T	G112D	11
TTN	NM_133378	Missense	chr2,179639029,T/C	Q2321R	10
TTN	NM_133378	Missense	chr2,179497327,G/T	A5404D	11
ZFAT	NM_020863	Missense	chr8,135577628,G/A	A975V	6
ZFAT	NM_020863	Missense	chr8,135612849,G/T	L769M	12

Supplementary Table 2. Genes sustaining multiple nonsynonymous SNVs in Barrett’s cases

Supplementary Table 3

Status	Short segment BE					long segment BE		Short segment BE			BE with LGD	
Patient ID	patient 1	patient 2	patient 3	patient 4	patient 5	patient 6	patient 7	patient 8	patient 9	patient 10	patient 11	patient 12
No. genes affected by amp												
BE Clone 1	0 (0)	4 (7)	8 (11)	0 (0)	0 (4)	10 (5)	6 (10)	1 (3)	8 (15)	57 (35)	5 (4)	2662 (821)
BE Clone 2	0 (2)	3 (3)	3 (6)	1 (3)	2 (4)	8 (4)	1(2)	0 (2)	11 (8)	2 (2)	7 (3)	2659 (778)
G Clone 1	8 (5)	2 (2)	3 (4)	0 (1)	3 (5)	3 (6)	1 (3)	3 (7)	5 (9)	2 (2)	1 (1)	12 (2)
G Clone 2	80 (13)	0 (0)	3 (5)	0 (0)	2 (4)	4 (6)	1 (2)	17 (10)	6 (10)	0 (2)	0 (0)	13 (3)
No. genes affected by del												
BE Clone 1	0 (3)	9 (12)	7 (8)	58 (119)	89 (20)	23 (11)	47 (9)	0 (1)	1 (1)	33 (18)	182 (54)	608 (156)
BE Clone 2	0 (2)	8 (8)	7 (5)	10 (9)	92 (22)	23 (11)	45 (6)	0 (4)	8 (11)	26 (8)	182 (53)	589 (111)
G Clone 1	1 (6)	1 (3)	0 (4)	1 (3)	187 (2)	2 (5)	0 (2)	0 (6)	1 (2)	0 (2)	0 (3)	0 (0)
G Clone 2	0 (4)	1 (1)	0 (4)	0 (1)	142 (20)	1 (8)	0 (2)	1 (6)	1 (1)	0 (2)	1 (4)	0 (1)

genes affected (No. CNVs)

Supplementary Table 3. Genes and CNV events in patient-matched BESC and GSC

Supplementary Table 4

Overlapped genes with Adenocarcinoma CNVs		Dulak AM et al., Cancer research 2012
BESC patient	Deletion	Amplification
Patient 1	No genes	No genes
Patient 2	PARD3B, FHIT, WWOX, CDKN2A	No genes
Patient 3	FHIT, WWOX, CDKN2A	No genes
Patient 4	FAM190A (1 clone), PTPRD, FHIT, CDKN2A	No genes
Patient 5	RUNX1, FHIT, WWOX, CDKN2A	No genes
Patient 6	PTPRD, FHIT, CDKN2A	No genes
Patient 7	FHIT, WWOX, CDKN2A	No genes
Patient 8	No genes	No genes
Patient 9	No genes	No genes
Patient 10	FHIT, WWOX, CDKN2A	No genes
Patient 11	PDE4D, , FHIT, WWOX,	No genes
Patient 12	SMAD4, PTPRD, PDE4D	ERBB2, FGFR1, GATA4, GATA6
	MACROD2, FHIT, WWOX, CDKN2A	KLF5, KRAS, MYB, MYC, SOX9, VEGFA

Supplementary Table 4. Genes affected by CNV in BESC and esophageal adenocarcinoma

Supplementary Table 5

Antigen	Company	Cat. No.	Dilution	link
p63	in house	-	1:100	
Krt5	Thermo Scientific / NeoMarkers	RM-2106-R7	1:500	http://www.thermoscientific.com/ecomm/servlet/productsdetail_11152_L11336_81817_11959831_-1
Sox9	Millipore	AB5535	1:1000	http://www.millipore.com/catalogue/item/ab5535
Cdh17	Sigma/HPA	HPA023614	1:100	http://www.proteinatlas.org/ENSG00000079112
Krt7	Leica	CK7 - 560	1:500	http://www.leicabiosystems.com/ihc-ish/novocastra-reagents/primary-antibodies/
Villin	Leica	NCL-L-Villin	1:100 or 200	http://www.leicabiosystems.com/ihc-ish/novocastra-reagents/primary-antibodies/
Sox2	Santa Cruz Biotechnology	sc-17320	1:50	http://www.scbio.de/datasheet-17320-sox-2-y-17-antibody.html
E-cadherin	Santa Cruz Biotechnology	sc-7870	1:100	http://www.scbt.com/datasheet-7870-e-cadherin-h-108-antibody.html
Gkn1	R&D systems	MAB6395	1:100	http://www.rndsystems.com/product_results.aspx?k=MAB6395
Cdx2	Biogenex	AM392-5M	ready-to-use	http://www.biogenex.com/index.php?page=shop.product_
Involucrin	Abcam	ab68	1:1000	http://www.abcam.com/involucrin-antibody-sy5-ab68.html
Involucrin	Leica Biosystems	NCL-INV	1:8000	http://www.leicabiosystems.com/ihc-ish/novocastra-reagents/primary-antibodies/details/product/involucrin-1/
Krt10	Covance	MMS-159S	1:50	https://store.crpinc.com/datasheet.aspx?Catalogno=MMS-159S
Gpa33	abcam	AB108938	1:1000	http://www.abcam.com/gpa33-antibody-epr4240-ab108938-references.html
Fabp1	Sigma	HPA028275	1:2500	http://www.sigmaaldrich.com/catalog/product/sigma/hpa028275?lang=en&region=US
Tff3	R&D systems	MAB4407	1:500	http://www.rndsystems.com/Products/MAB4407
Krt20	Dako	M7019	1:500	http://www.dako.com/us/ar38/p103700/prod_products.htm
Reg4	R&D systems	AF1379	1:500	http://www.rndsystems.com/product_results.aspx?k=AF1379
Mucin 2	Santa Cruz Biotechnology	sc-15334	1:100 or 400	http://www.scbt.com/datasheet-15334-mucin-2-h-300-antibody.html
Pgc	Sigma	HPA031718	1:200	http://www.sigmaaldrich.com/catalog/product/sigma/hpa031718?lang=en&region=US

Supplementary Table 5. List of antibodies
